# Aging: The Fading Signal of Natural Selection

**DOI:** 10.3389/fgene.2012.00155

**Published:** 2012-08-28

**Authors:** Lee F. Greer

**Affiliations:** ^1^Department of Ecology and Evolutionary Biology, University of CaliforniaIrvine, CA, USA

The surprising discovery that age-specific mortality rates (and fecundity rates) plateau in late life, in contrast to the traditional Gompertz prediction of the acceleration of age-specific mortality rates, has motivated Rose and his collaborators to seek explanations grounded in evolutionary theory, such as the decline of natural selection with age predicted by Hamilton in 1966 (Mueller et al., 2011). From this approach has emerged Rose’s stated idea that aging is the deterioration or detuning of adaptation with age. To push the musical metaphor, as the forces of natural selection attenuate, the coherent tuned signal of adapted allele frequencies fades until lost in the static noise of random genetic drift. The long-lived fly experiments in the Rose laboratory which indicate that adjusting the timing of the antagonistic pleiotropy between reproduction and survival in turn adjusts the timing of the late life plateaus comprise a most striking confirmation of the evolutionary approach.

Despite these predictive successes, aging continues to be almost reflexively thought of by many as the inexorable accumulation of cellular and physiological damage and wear with age. Even researchers who know of the decline of Hamilton’s forces have often assumed, as Rose points out, that this decline fits with the inexorable damage accumulation thesis. This old thinking casts a shadow not only on much aging research but its application in traditional pharmaceutical approaches to the “diseases of aging” and in clinical gerontology.

Now in the age of genomics, we can test aspects of the model with rich new data sets. In comparing the genomes of long-lived flies with flies of ordinary life span, it would be fascinating to search for altered expression levels (*via* microarray studies as J. L. G. suggests in his commentary on “What is Aging?”) in genes at succeeding stages in life history. Another potentially illuminating suite of tests would entail the use of varied phylogenetic methods (Suzuki, 2010) to detect differences between lineages in the strength of selection versus nearly neutral genetic drift in those genes with altered expression and across regions of the genomes of long-lived and ordinary flies.

If the age of genomics has taught us anything, it is that genomic adaptations are likely to be slight and additive across numerous genes and their regulatory regions.
